# Exploring the interplay of emotional intelligence, psychological resilience, perceived stress, and life satisfaction: A cross-sectional study in the Turkish context

**DOI:** 10.1186/s40359-024-01860-0

**Published:** 2024-06-21

**Authors:** Aslı Kartol, Servet Üztemur, Mark D. Griffiths, Derya Şahin

**Affiliations:** 1 Department of Psychological Counseling and Guidance, Faculty of Education, Trakya University, Edirne, Türkiye; 2 Department of Social Studies Education, Faculty of Education, Anadolu University, Eskişehir, Türkiye; 3 International Gaming Research Unit Psychology Department, Nottingham Trent University, Shakespeare Street, Nottingham, England; 4 Department of Fine Arts, Faculty of Education, Inonu University, Malatya, Türkiye

**Keywords:** Emotional intelligence, Life satisfaction, Perceived stress, Resilience

## Abstract

**Background:**

Emotional intelligence and life satisfaction are essential components for good psychological well-being. Studies examining the elements contributing to emotional intelligence and its relationships with different psychological constructs are likely to positively contribute to mental health. Therefore, the present study examined the mediating roles of perceived stress and psychological resilience in the relationship between emotional intelligence and life satisfaction.

**Methods:**

The study sample comprised 780 university students (62.3% females) studying at universities in different regions of Türkiye. An online survey included the Emotional Intelligence Scale, Satisfaction with Life Scale, Psychological Resilience Scale, and Perceived Stress Scale. A multifactorial complex predictive correlational design was used.

**Results:**

The results showed that emotional intelligence was (i) positively correlated with life satisfaction and psychological resilience, and (ii) negatively correlated with perceived stress. In the final model, perceived stress and psychological resilience played a mediating role in the relationship between emotional intelligence and life satisfaction. The findings suggest that higher emotional intelligence may lower perceived stress and appears to have a positive effect in relation to life satisfaction and psychological resilience.

**Conclusion:**

Individuals working in the field of mental health need to help individuals increase their level of EI, which may help reduce the level of perceived stress and increase psychological resilience and life satisfaction.

## Introduction

Individuals with high emotional intelligence (EI) are better able to regulate emotions and are more capable of coping, maintaining interpersonal relationships, and adapting to the environment [1, 2]. Moreover, research conducted in March 2020 reported that approximately 2.66 million scientific studies had mentioned EI [1]. Considering that EI increases personal success [3], it is necessary to develop EI skills at an early age [4]. EI-based interventions have been found to increase life satisfaction and resilience [5]. Kotsou et al. [6] showed that EI-based interventions are associated with high life satisfaction and low stress. Schutte et al. [7] found that EI-based interventions have positive results on mental and physical health, social relationships, and work performance. High EI has also been associated with lower levels of perceived stress [8, 9]. Ciarrochi et al. [10] claimed that EI helps individuals to protect themselves against the undesirable effects of stress and therefore to cope with depression, anxiety, and hopelessness.

EI also contributes positively to individual differences in physiological functioning, emotion regulation, coping with life events, establishing supportive relationships, and health behaviors [11]. Due to the trainable nature of social and emotional skills [12], factors associated with EI are more likely to increase life satisfaction. A meta-analysis found that emotional intelligence is associated with better health [13]. It has also been found that emotional intelligence is associated with a more meaningful life and positive well-being [14]. Accepting and managing emotions, rather than ignoring them, is a skill that enhances personal well-being and has a direct positive impact on wellbeing [15]. The aim of the present study was to examine the mediating roles of perceived stress and psychological resilience in the relationship between emotional intelligence and life satisfaction.

### Emotional intelligence and life satisfaction

Life satisfaction is a construct at the core of positive psychology studies and is an important component of mental health in psychological, behavioral, social, and interpersonal domains [16]. Although life satisfaction is related to quality of life, not every person with good quality of life may be satisfied with life. This satisfaction is closely related to how much individuals like the life they live, that is, how positively they evaluate their current life [17]. Life satisfaction is the result of positive life experiences and contributes directly to psychological resilience [18]. Blasko-Belled et al. [19] found that emotional intelligence is directly related to well-being and happiness.

Stressful and traumatic events may affect well-being in both a long-term and direct way [20]. Moreover, some skills that individuals have (e.g., resilience, life satisfaction, emotional intelligence), especially in difficult life events, can play a protective role. Negative life events are an inevitable reality. Galindo-Dominguez, Iglesias [21] found that emotional intelligence and life satisfaction were protective factors between victimization and suicidal ideation. Another study showed that emotional intelligence is an effective skill in reducing suicidal ideation [22]. Diener [23] characterized life satisfaction as the cognitive component of subjective well-being. Fortunately, it is possible to increase life satisfaction with psychological interventions [24]. Moreover, EI is also associated with a more positive mood [1]. Emotional intelligence has been consistently linked to higher life satisfaction, with studies indicating a strong positive correlation between EI and life satisfaction [25–28]. It is important to address these components for a more meaningful and positive life.

### Emotional intelligence, life satisfaction, resilience, and perceived stress

Psychological resilience is the ability of individuals and communities to adapt successfully in the face of adverse situations. It can develop the individual as opposed to escaping or avoiding psychopathology. Studies examining psychological resilience have increased [29, 100, 101]. When talking about resilience, this often refers to the presence of a situation that needs to be returned or coped with. Many studies have tried to determine the factors that reduce or increase resilience [30]. Since the early 2000s, psychological resilience has been the subject of intensive and increased research in the field of positive psychology [31, 98, 99]. Moreover, Masten [32] has used the term ‘ordinary magic’ for resilience and emphasized that this skill exists in every individual and that it has a protective effect in difficult life conditions and unexpected negative situations.

The effects of stress on physical and emotional health in human life can be deep and destructive. So much so, that this stress has been characterized by World Health Organization (WHO) as the “health epidemic of the 21st century”. Stress is a component whose perception varies from person to person and progresses in coordination with the flexibility and resilience of individuals [33]. Stress is a highly subjective phenomenon as well as being caused by external conditions and experiences. The interaction between an individual’s perception of the resources they have, and external demands is an important determinant of stress. It is a subjective reaction to an objective situation [34, 104].

Exposure to too much stress can have negative outcomes, and not experiencing stress at all is not a positive situation in terms of health [102]. Low amounts of stress motivate and activate the individual [35]. Reducing perceived stress levels and increasing adaptive coping are critical for stress management [33–36]. Determining the role of stress in problem situations is important in physical and psychological terms [37]. Moreover, it has been shown that EI (i) significantly predicts perceived stress [38], and (ii) increases stress resilience [39]. Therefore, examining the mediating roles of perceived stress and resilience in the relationship between emotional intelligence and life satisfaction will likely contribute to the extant literature regarding well-being.

### Student well-being

Recently, there has been an increase in studies focusing on students’ well-being [40]. Educational research has focused on developing students’ strengths and abilities rather than their weaknesses [20]. Shankland and Rosset [41] highlighted the importance of positive psychology-based interventions that promote well-being in education. Well-being is considered very important for students to be able to perform at school and to be protected from self-destructive stressors [42]. Pishghadam et al. [43] found that emotional intelligence is an important predictor of academic success. Moreover, psychological resilience is a very important construct for students, both in terms of academic success and social life [44]. Therefore, it is important to focus on mechanisms such as emotional intelligence, well-being, and life satisfaction that enable young people to adapt to the changes they may encounter in their lives [45]. For this reason, the present study group comprised university students.

### The present study

Individuals with high EI have higher life satisfaction [25–27], are more psychologically resilient [39, 46, 47], and have lower stress perception tendencies [38, 48–50]. Although studies have shown that there is a significant relationship between emotional intelligence and life satisfaction [25, 51], research in the past decade has focused on investigating the mechanisms that mediate the relationship between these two variables [20, 52–54]. Emotional intelligence has been the subject of many studies in psychology, as well as in areas such as business and education. In Türkiye, where the present study was carried out, studies based on EI have mostly focused on job satisfaction and performance characteristics of this skill [55–58]. Studies examining the mental health of university students have become an increasing public health problem [59]. Untreated mental health problems not only reduce the academic performance of university students but also pose a risk for every student in the campus environment [60]. Therefore, the present study purposefully recruited university students as a sample because they are an at-risk group.

**Fig. 1:**
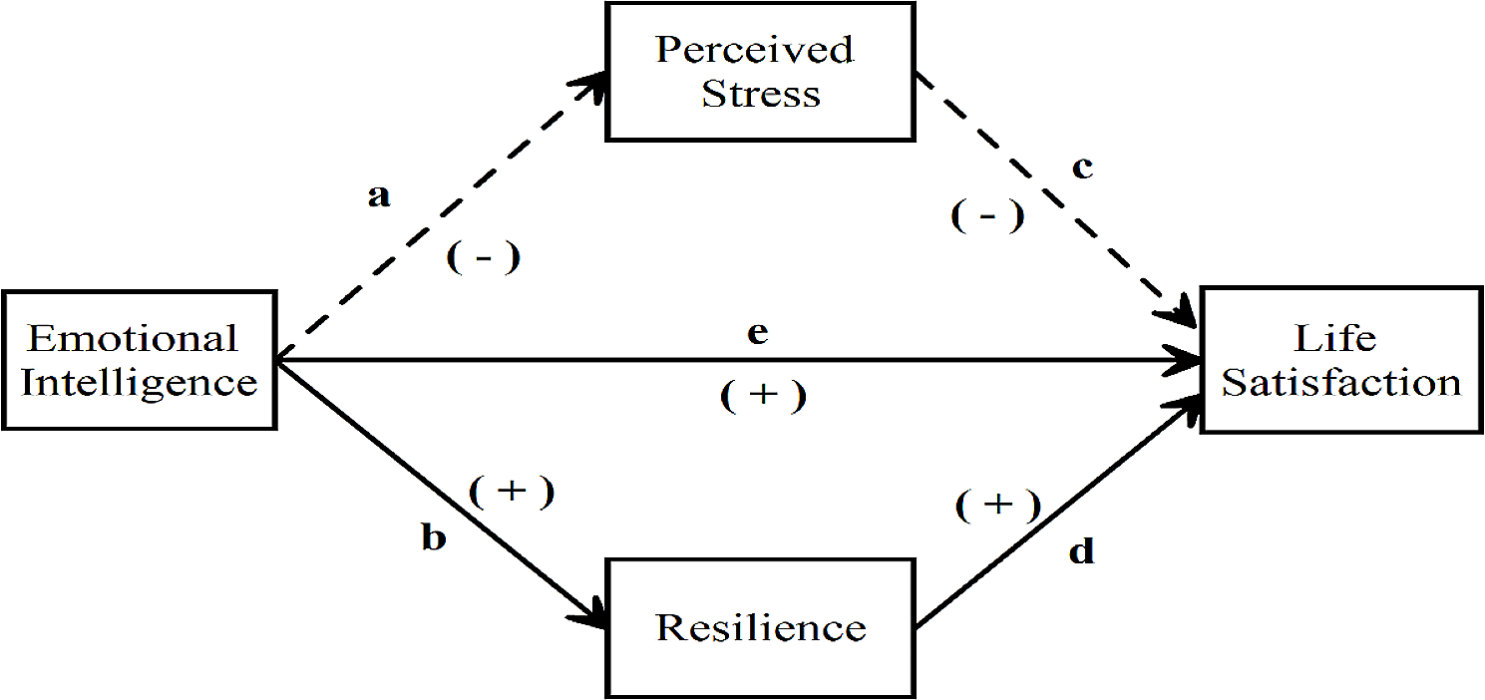
Relationships between emotional intelligence, resilience, perceived stress, and life satisfaction

Since the concept of EI includes the concept of ‘intelligence’, this intelligence modelling dimension is a skill that can be developed and comes from modelling. Moreover, it can be developed with EI intervention programs. In this context, it is thought that studies on the elements underlying this skill and its relationships with different concepts will make positive contributions to mental health outside of work life and will guide the studies to be carried out by health professionals studying in this field. In the present study, it was hypothesized that resilience and perceived stress may play a mediating role in the relationship between EI and life satisfaction. Based on the assumption that psychological resilience and perceived stress have mediating effects on the relationship between EI and life satisfaction, a model that hypothesized the relationships between these four variables was created based on the relevant literature and is shown in Fig. 1.

Increasing emotional intelligence levels and life satisfaction have significant contributions to individuals’ well-being. In this respect, although there are studies examining the association between these variables, there are few studies investigating the underlying factors that may affect this association. Studies conducted to increase well-being are very important for individual and social welfare. Moreover, psychological resilience is an important component of well-being [61]. Seligman [62], one of the founders of positive psychology, emphasized the importance of studies that increase well-being such as subjective well-being and life satisfaction, and emphasized that individuals should focus on their strengths. Eldeleklioglu and Yildiz [63] reported that there are positive relationships between the expression of emotions, resilience, and well-being. Stressful events have always existed and will continue to exist in people’s lives (e.g., the COVID-19 pandemic) [103]. It is important to make preventive interventions for individuals’ mental health for future studies. Consequently, in the present study, the mediating roles of perceived stress and resilience in the relationship between emotional intelligence and life satisfaction were examined.

The notion that perceived stress and resilience may affect the relationship between emotional intelligence and life satisfaction was tested in the present study. Previous studies have examined the relationships between emotional intelligence, life satisfaction, resilience, and perceived stress separately. However, no previous study has ever examined how emotional intelligence may affect life satisfaction through perceived stress and resilience. Moreover, there are few studies in Türkiye. Therefore, the present study addressed this cultural gap. In line with the hypothetical model in Fig. 1, the following research question (RQ) was addressed and six hypotheses (Hs) were proposed:

- RQ: How and in what direction are there relationships between these four aforementioned variables?
- H_1_: Emotional intelligence will be positively associated with life satisfaction.
- H_2_: Emotional intelligence will be negatively associated with perceived stress.
- H_3_: Emotional intelligence will be positively associated with resilience.
- H_4_: Perceived stress will be negatively associated with life satisfaction.
- H_5_: Resilience will be positively associated with life satisfaction.
- H_6_: In the model tested, perceived stress and resilience will have a mediating effect between emotional intelligence and life satisfaction.

## Method

### Research design

In the present study, a multifactorial complex predictive correlational design was used to investigate the structural relationships between university students’ EI, psychological resilience, perceived stress, and life satisfaction. In predictive correlation studies, the relationships between variables are examined and the other is predicted based on one of the variables. Multifactorial predictive correlational designs are intended to test only direct relationships or both direct and indirect relationships [64]. In the present study, (i) EI was identified as the predictor variable, (ii) perceived stress and resilience as mediating variables, and (iii) life satisfaction as the predicted variable.

### Participants

The participants (62.3% female) were 780 university students (37% health sciences, 32% education sciences, 17% science, and 14% fine arts) from seven different regions of Türkiye using a convenience sampling technique. To ensure maximum diversity, care was taken to select participants from as many different universities and faculties as possible. The grade levels of the participants were as follows: freshman 24.1%; sophomore 18.3%; junior: 21.9%; senior 35.6%). The study procedures were designed in line with the Declaration of Helsinki guidelines. Informed consent was obtained from all participants.

### Procedure and ethics

To collect data from university students in different universities in Türkiye, a 45-item online survey was created. Data collection was carried out online using *Google Forms*. Türkiye consists of seven regions. To increase the diversity of participants, academics working at the largest university in each region were contacted. University students were recruited to participate in the survey through the contacted academics. The data were only collected voluntarily from participants. There were no missing data because surveys could not be submitted unless all data fields had been completed. Ethical approval was granted by the Gaziantep University Ethics Committee (Ethics Number: 291,500).

### Measures

#### Emotional Intelligence Scale Short Form (EISSS)

The EISSS [65, 66] was used to assess emotional intelligence. The scale comprises four sub-dimensions (well-being, self-control, emotionality, sociability), each consisting of four items (e.g., *“I have a tendency to change my decisions frequently”*) assessed using a seven-point Likert-type scale ranging from 1 (*I never agree*) to 7 (*I completely agree*). Higher scores indicate greater EI competence. Confirmatory factor analysis (CFA) was conducted to ensure construct validity. Results confirmed the original four-factor structure of the scale and the fit indices were at an acceptable level: χ^2^/df = 5.97, incremental fit index (IFI) = 0.91, root mean square error of approximation (RMSEA) = 0.07, standardized root mean squared residual (SRMR) = 0.05, comparative fit index (CFI) = 0.91, Tucker-Lewis coefficient (TLI) = 0.90 [67]. In the present study, the overall scale’s internal consistency coefficient (Cronbach alpha) was 0.84.

#### Psychological Resilience Scale Short Form (PRSSS)

The PRSSS [68, 69] was used to assess resilience. The single dimension scale comprises six items (e.g., *“I can recover myself quickly after troubled times”*) assessed on a five-point Likert scale ranging from 1 (*not suitable for me at all*) to 5 (*totally appropriate*). Higher scores indicate increased resilience. In the scale, Items 2, 4, and 6 are reverse-coded. CFA results confirmed the original one-factor structure of the scale, and the fit indices were at an acceptable level: χ^2^/df = 1,283, SRMR = 0.01, IFI = 0.99, RMSEA = 0.02, TLI = 0.99, CFI = 0.99. In the present study, the scale’s internal consistency coefficient (Cronbach alpha) was 0.82.

#### Perceived Stress Scale (PSS)

The PSS [70, 71] was used to assess perceived stress. The scale comprises two sub-dimensions (perception of inadequate self-efficacy, perception of stress) each consisting of seven items (e.g., *“In the last month, how often have you felt nervous and “stressed”?*) assessed on a five-point Likert-type scale ranging from 0 (*never*) to 5 (*very often*). Higher scores indicate greater perceived stress. Seven of the scale items are reverse-coded. As a result of CFA, two items with low factor loadings were removed from the analysis. The analysis was repeated with the remaining items and the original two-factor structure of the scale was confirmed and the fit indices were at an acceptable level: χ^2^/df = 4.192, IFI = 0.95, RMSEA = 0.06, SRMR = 0.06, CFI = 0.95, TLI = 0.94. In the present study, the internal consistency coefficients (Cronbach alphas) of the sub-dimensions were 0.85 and 0.81, respectively.

#### Life Satisfaction Scale (LSS)

The LSS [23, 72] was used to assess life satisfaction. The single-dimension scale comprises five items (e.g., *“I have a life close to my ideals”*) in a single dimension and five-point Likert type ranging from 1 (*strongly disagree*) to 5 (*strongly agree*). Higher scores indicate greater life satisfaction. CFA results confirmed the original one-factor structure of the scale, and the fit indices were at an acceptable level: χ^2^/df = 4,395, SRMR = 0.01, IFI = 0.99, RMSEA = 0.06, TLI = 0.98, CFI = 0.99. The scale’s internal consistency coefficient (Cronbach alpha) was 0.84 in the present study.

### Data analysis

In the first stage of analysis, the standardized z-scores of the variables were assessed to identify any potential outliers. It was found that all standardized z-scores fell within the acceptable range of -3 to + 3, indicating the absence of any one-way outliers within the dataset [96]. Furthermore, the Variance Inflation Factors (VIF) were examined to evaluate the presence of multicollinearity among the predictor variables of the dependent variable (emotional intelligence: 1.28, perceived stress: 1.42, resilience: 1.14). Since these values are between 1 and 5, it can be said that there is no multicollinearity problem between the variables [97]. In the second stage, analysis was used to determine whether there was a common method bias (CMB) problem by using explanatory factor analysis and applying Harman’s one-factor test to a total of 45 items related to the four scales. In line with the unrotated principal component factor analysis, a total of eight factors were obtained with eigenvalues higher than 1. Since the variance of the first factor (25.15%) was less than 40% in the dataset where 56.86% of the total variance was explained, it can be said that CMB did not constitute a significant problem among the relevant variables [73]. In the second stage, descriptive statistics were used. In the third stage, Pearson product-moment correlation coefficients were calculated to determine the relationships between the variables. In the fourth stage, hierarchical regression analysis was used to evaluate the mediating effects of resilience and perceived stress in the relationship between EI and life satisfaction. To check whether there was a multicollinearity problem, further analysis checked whether the tolerance values were greater than 0.20 and the variance inflation factor (VIF) was less than 10. For multivariate normality assumptions, it was tested whether the skewness and kurtosis values were between − 1 and + 1. Since the data were collected online, there were no missing values in the dataset [74] because the survey could not be submitted if there were missing responses. In addition to hierarchical regression analyses, the bias-corrected bootstrapping method was applied as suggested by Hayes [75] with the SPSS *Process Macro* plug-in to test the statistical significance of the mediating effect of resilience and perceived stress in the relationship between EI and life satisfaction. The number of samples was increased to 5,000 by random sampling method to create 95% confidence intervals. The absence of a zero value between confidence intervals indicates that the mediating effect tested in the model was statistically significant [75]. SPSS 25 was used for all analyses, with a significance level of *p* < .05.

## Results

### Descriptive statistics and correlations

Descriptive statistics and correlation values related to EI, resilience, perceived stress, and life satisfaction are shown in Table 1.

**Table 1:** Descriptive statistics and correlation analysis (*N* = 780)

Variable	Mean	SD	Skewness	Kurtosis	(1)	(2)	(3)	(4)
1. Emotional intelligence	4,71	0.91	− 0.150	− 0.425	1	,562^**^	-,587^**^	,445^**^
2. Resilience	3,02	0.64	− 0.107	− 0.056		1	-,648^**^	,404^**^
3. Perceived stress	3,07	0.64	0.452	0.596			1	-,431^**^
4. Life satisfaction	2,94	0.91	− 0.077	− 0.537				1

**p < .01

As can be seen in Table 1, there was a moderate positive correlation between EI and life satisfaction. EI had a positive correlation with resilience and a negative correlation with perceived stress. As expected, perceived stress and other variables had moderately significant negative correlations. Considering the skewness and kurtosis values, it can be said that the dataset is normally distributed (kurtosis and skewness ≤ |1|) [67].

### Mediation analyses

A four-stage hierarchical regression analysis was conducted to test the mediating effects of resilience and perceived stress in the relationship between EI and life satisfaction. In the first stage, the dependent variable (life satisfaction) was predicted by the independent variable (EI). In the second stage, the mediating variable (resilience) was predicted by the independent variable. In the third stage, the mediating variable (perceived stress) was predicted by the independent variable. In the fourth stage, the dependent variable was predicted by both predictor and mediator variables. In the literature, it has been reported that emotional intelligence [76, 77], perceived stress [78, 79], resilience [80] and life satisfaction [81] differ significantly according to gender. Therefore, gender was included in all analyses as a control variable. The results of the four-stage hierarchical regression analysis are shown in Table 2.

**Table 2:** Hierarchical regression analysis predicting life satisfaction

Regression equation	Dependent variable	Independent variable	B	β	*t*	*R* ^2^	Δr^2^	ΔF
1	Life Satisfaction	Gender	0.080	0.043	1,326	0.20	-	97,154
		Emotional Intelligence	0.443	0.444	13,811^***^			
2	Perceived Stress	Gender	− 0.052	− 0.039	-1,348	0.346	-	205,867
		Emotional Intelligence	− 0.414	− 0.585	-20,175^***^			
3	Resilience	Gender	0.004	0.002	0.084	0.315	-	179,221
		Emotional Intelligence	0.543	0.561	18,913^***^			
3	Life Satisfaction	Gender	0.065	0.034	1,118	0.253	0.053	65,629
		Emotional Intelligence	0.255	0.255	6,362^***^			
		Perceived Stress	− 0.268	− 0.190	-4,354^***^			
		Resilience	0.141	0.136	3,207^***^			

***p < .001

**Fig. 2:**
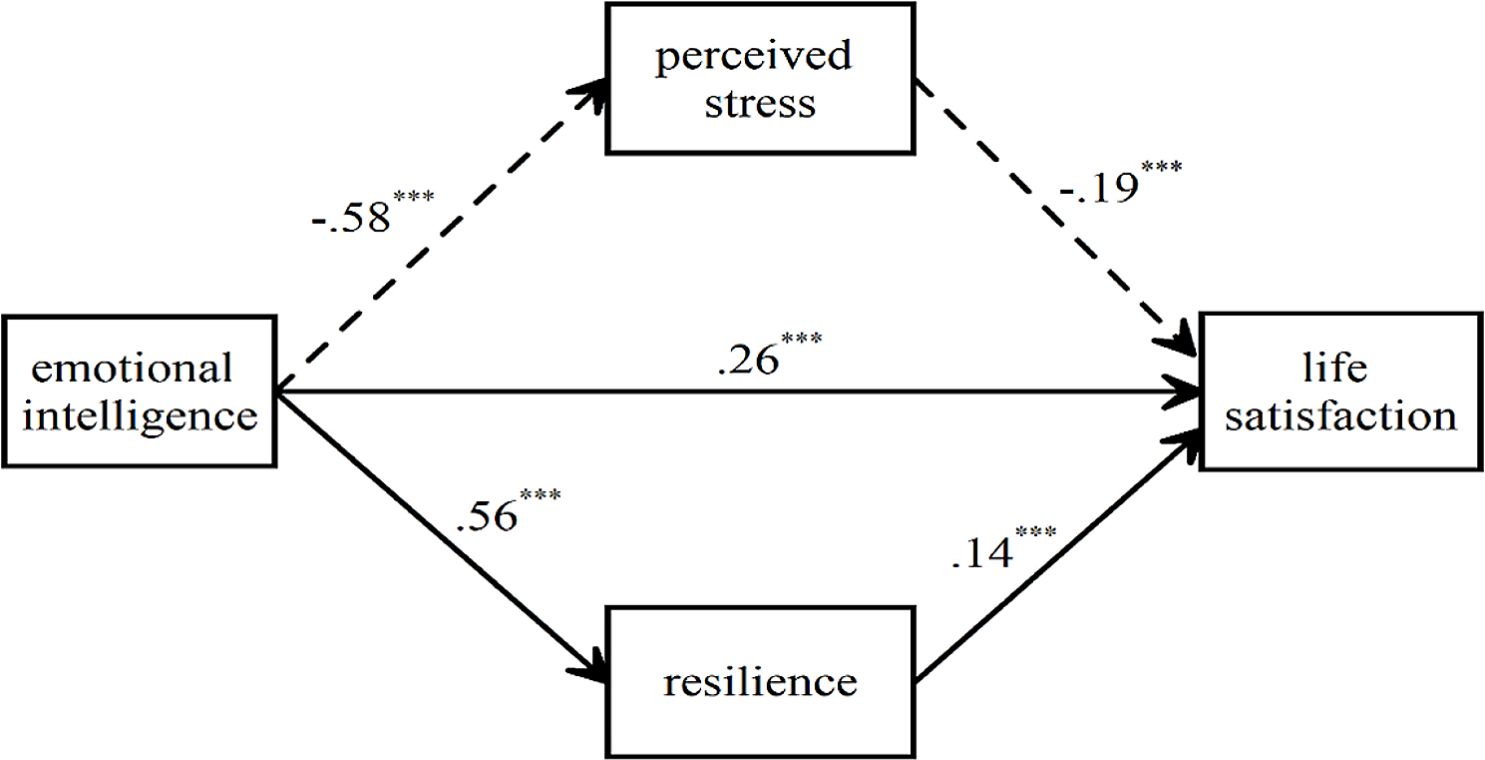
Standardized beta coefficients for the mediating effect of resilience and perceived stress on the relationship between emotional intelligence and life satisfaction (*N* = 780). ^***^p < .001

As can be seen in Table 2, in the first stage (Regression Equation 1), a direct path was established between EI and life satisfaction (H_1_) and the independent variable (EI) predicted the dependent variable (life satisfaction) significantly and positively (β = 0.44, *t* = 13,811, *p* < .001). According to these findings, H_1_ was confirmed and 20% of the variance in life satisfaction was explained by EI. In the second stage (Regression Equation 2), EI predicted perceived stress negatively and H_2_ was accepted (β=-0.58, *t*=-20,175, *p* < .001). Accordingly, 35% of the variance in perceived stress was explained by EI. In the third stage (Regression Equation 3), EI predicted resilience positively and H_3_ was accepted (β = 0.56, *t* = 18,913, *p* < .001). Accordingly, 32% of the variance in resilience was explained by EI. In the fourth stage (Regression Equation 4), after the mediating variables were included in the model, although EI predicted life satisfaction significantly, the effective coefficient of EI decreased (β = 0.25, *t* = 6,362, *p* < .001). According to these findings, resilience and perceived stress partially mediated the relationship between EI and life satisfaction, and H_4_ and H_5_ were confirmed. The mediating variable analyses are shown in Fig. 2.

According to Fig. 2, the indirect effect of EI on life satisfaction through resilience and perceived stress (0.58 × 0.19 = 0.11; 0.56 × 0.14 = 0.08; 0.11 + 0.08 = 0.19) corresponds to 42% (0.19 ÷ 0.45 = 0.42) of the total effect (0.26 + 0.19 = 0.45). With the addition of mediating variables to the model, there was an increase in the variance explained in the dependent variable at the 0.05 level. In addition, 25.3% of the variance in the dependent variable was explained by the independent variable, mediator variables, and control variables together. When mediator variables were not included in the model, this rate decreased to 20%. It was observed that the control variable (gender) had no significant effect on the dependent variable and mediator variables. To test the significance of the mediating effects of robustness and perceived stress, the ‘bias-corrected bootstrapping’ method was applied with the SPSS *Process Macro* plug-in suggested by Hayes [75]. Indirect effect coefficients and 95% confidence intervals are shown in Table 3.

**Table 3:** The bootstrapping for the partial mediation model (*N* = 780)

	Model Paths	SPC	SE	95% Confidence Interval	
				Lower	Upper
**Standardized Indirect**	EI→RES→LS	0.08^**^	0.02	0.13	0.25
	EI→PS→LS	0.11^**^	0.02	0.06	0.17

EI = Emotional Intelligence, RES = Resilience, PS = Perceived Stress, LI = Life Satisfaction, SPC = Standardized path coefficient, SE = Standard error. ^**^p < .01,

As can be seen in Table 3, after 5,000 bootstrap procedures, the indirect path coefficient of resilience was significant and there was no zero between confidence intervals (bootstrap coefficient = 0.08, 95% confidence interval = 0.13-0.25). The indirect path coefficient of perceived stress was significant and there was no zero between confidence intervals (bootstrap coefficient = 0.11, 95% confidence interval = 0.06-0.17). According to these findings, H_6_, which hypothesized that resilience and perceived stress would mediate the relationship between EI and life satisfaction, was confirmed.

## Discussion

In the present study, the relationships between emotional intelligence (EI), life satisfaction, resilience, and perceived stress were analyzed and the mediating role of psychological resilience and perceived stress in the relationship between EI and life satisfaction was tested. The first finding of the present study was that EI positively predicted life satisfaction (supporting H_1_). This finding has been supported in many studies [25–28]. Azpiazu et al. [82] found that emotional clarity and emotional repair contribute to the well-being of adolescents. Quality of life and emotional adjustment are related because if emotions are not managed effectively, stressors could lead to mental health problems, influencing EI (attention, clarity, and repair) [83].

The second finding was that EI had a significant negative relationship with perceived stress (supporting H_2_). This finding is also supported in the literature [38, 48–50]. In their study of students, Karaoglan Yılmaz et al. [84] found that the higher the emotional intelligence level, the lower their perceived stress level. Based on these findings, individuals with high EI are less affected by stressful situations. It can be said that the stress management mechanism, which is a sub-dimension of EI, reduces the perceived stress level. The third finding was that EI positively predicted resilience (supporting H_3_). Again, this finding has been shown in many studies [39, 46, 47]. Self-awareness, good interpersonal relationships, and the ability to cope with negative events contribute positively to resilience and increase well-being. In their study of adolescents, Collado-Soler et al. [45] found that emotional intelligence and psychological resilience were strongly related and emphasized that young people should develop a series of internal resources that serve as protection against the changes they may encounter in their lives and facilitate their adaptation.

The fourth finding in the present study was that perceived stress significantly and negatively predicted life satisfaction (supporting H_4_). There are supportive studies in the literature [85–87]. The way stress is perceived affects the level of satisfaction. The way of perceiving negative life events affects life satisfaction. As the level of perceived stress increases, psychological problems may be inevitable. The fifth finding was that resilience positively predicted life satisfaction (supporting H_5_). Many studies have found that as the level of psychological resilience of individuals increases, their satisfaction with life increases [88–90]. The final finding of the present study was that perceived stress and resilience played mediating roles in the relationship between EI and life satisfaction (supporting H_6_). It has been shown that individuals with high levels of EI have low levels of perceived stress, which increases psychological resilience, and therefore life satisfaction is higher.

In the present study, two paths were considered in the relationship between EI and life satisfaction. In the first path, perceived stress mediated the relationship between EI and life satisfaction. Looking at the literature, studies have shown that perceived stress has a mediating effect between EI and life satisfaction [91, 92]. In the second path, resilience mediated the relationship between EI and life satisfaction. There are also studies in which psychological resilience has played a mediating role between EI and life satisfaction [18, 61, 93].

It has previously been observed that individuals with high EI can establish healthier relationships with themselves and their environment and cope with stress more easily [94, 95]. Satisfaction from life takes shape depending on the experiences of the individual. Negative life events especially show these individual coping skills and cause individuals to adapt more easily than others. Stressful events and negative life experiences are shaped by the way individuals perceive these situations. In this respect, managing stress is a very important skill. In the present study, emotional intelligence appeared to affect life satisfaction and emotional intelligence appeared to affect life satisfaction through perceived stress and psychological resilience. Therefore, the importance of emotional intelligence for a more fulfilling life appears to have been confirmed. All these components (emotional intelligence, life satisfaction, and resilience) appear to provide evidence for a more satisfying life and greater well-being.

### Limitations

There are some limitations in the present study. First, the findings of the study were based on self-report data which can be subject to various methods biases (e.g., social desirability). Second, all the participants were Turkish university students recruited using convenience sampling, so the sample was not representative of Turkish university students or the Turkish population. Moreover, the sample had a higher proportion of females (62.3%), which may have potentially influenced the results. Fourth, due to the cross-sectional nature of the study, causal relationships between emotional intelligence and life satisfaction could not be determined. Although the mediation analyses were consistent with theoretical predictions, caution should be exercised in drawing causal conclusions because the mediation design used in the study was correlational. Causality regarding emotional intelligence can only be investigated through longitudinal and experimental studies. Fifth, the study’s data were collected from a modest number of participants. Therefore, it is difficult to generalize the results to larger populations.

### Implications

Despite the limitations, the fact that emotional intelligence is a skill that can be developed through education makes the present study’s findings important. The findings can be used by psychologists, mental health professionals, educational practitioners, and counsellors to help young people to develop emotional intelligence skills in educational and therapeutic settings. To live a more satisfying life, individuals need to control their emotions, have healthy relationships, and have an adaptive social skills. In this regard, the designing of individual and group interventions to improve students’ emotional intelligence levels will likely make a significant contribution to the well-being of students. Emotional intelligence-based studies and psychoeducational practices are likely to be effective in improving the well-being of individuals by positively influencing their social and emotional skills and attitudes.

## Conclusion

The results showed that perceived stress and psychological resilience played a mediating role in the relationship between emotional intelligence and life satisfaction. Although it is known that there is a direct relationship between emotional intelligence and life satisfaction, it is important to investigate other psychological factors that may influence this relationship. The present study investigated variables that have never been previously investigated simultaneously before. Studies on the factors that influence life satisfaction are important to help improve the mental health of individuals. The study was also carried out among a Turkish sample, which again (and taking into account cultural differences), has never been done before.

## Data Availability

The original form and data of this study are available from the corresponding author upon reasonable request.

## Abbreviations

EI: Emotional Intelligence
EISSS: Emotional Intelligence Scale Short Form
LSS: Life Satisfaction Scale
PRSSS: Psychological Resilience Scale Short Form
PSS: Perceived Stress Scale
